# Small-Molecule CCR4 Antagonists in Cutaneous T-cell Lymphoma

**DOI:** 10.1158/2767-9764.CRC-24-0297

**Published:** 2024-10-22

**Authors:** José S. Enriquez, Xiaohong Wang, Loka Reddy Velatooru, Wei Han, Pedram Bijani, Xiao Ni

**Affiliations:** Department of Dermatology, The University of Texas MD Anderson Cancer Center, Houston, Texas.; Department of Dermatology, The University of Texas MD Anderson Cancer Center, Houston, Texas.; Department of Dermatology, The University of Texas MD Anderson Cancer Center, Houston, Texas.; Department of Dermatology, The University of Texas MD Anderson Cancer Center, Houston, Texas.; Department of Dermatology, The University of Texas MD Anderson Cancer Center, Houston, Texas.; Department of Dermatology, The University of Texas MD Anderson Cancer Center, Houston, Texas.

## Abstract

Mycosis fungoides (MF) and Sézary syndrome (SS) are two most common types of cutaneous T-cell lymphoma (CTCL). Despite advances in understanding the pathogenesis of MF and SS, effective treatments remain limited. CC chemokine receptor-4 (CCR4) is highly expressed on CTCL cells and serves as a great therapeutic target. Mogamulizumab, an FDA-approved anti-CCR4 antibody, has shown efficacy in treating MF/SS; however, its side effects have raised concerns, underscoring the need for more effective and less toxic CCR4-targeted therapies. Although small-molecule CCR4 antagonists have been studied in other diseases involving CCR4^+^ Th2 cells and regulatory T cells, their effects in CTCL have not been previously explored. This study assessed the effects of two small-molecule CCR4 antagonists: C021 (class-I) and AZD-2098 (class-II) in MF-derived cell line (MJ) and SS-derived cell line (HuT 78) *in vitro* and *in vivo*. As per results, both C021 and AZD-2098 inhibited chemotactic responses to CCL17 and CCL22 in MJ and HuT 78 cells. However, only C021 downregulated CCR4 expression, inhibited cell proliferation, induced cell apoptosis and cell-cycle arrest, and decreased colony formation in MJ and HuT 78 cells *in vitro*. Furthermore, only C021 inhibited tumor growth in CTCL xenograft mice *in vivo*. These findings suggest that class-I CCR4 antagonists such as C021 exert more potent antitumor effects on CTCL cells *in vitro* and *in vivo* than do class-II CCR4 antagonists like AZD-2098, highlighting their potential for clinical application.

**Significance:**

Our findings are of interest to readers because they bring new evidence that small-molecule CCR4 antagonists may be an alternative therapeutic strategy to target CCR4^+^ CTCL cells. They may inhibit CCR4 function but not eradicate cells, so the side effects may be avoided or minimized.

## Introduction

Cutaneous T-cell lymphoma (CTCL) is a non–Hodgkin lymphoma that initiates in the skin and can involve the blood. Mycosis fungoides (MF) and Sézary syndrome (SS) are the most common subtypes of CTCL. Although recent research advances help our understanding of the pathogenesis of MF/SS, effective treatments are still rare. CC chemokine receptor-4 (CCR4), a G protein–coupled receptor, is mainly expressed on type-2 helper T cells (Th2), regulatory T cells (Treg), and skin-homing T cells. Studies have found that malignant T cells in MF/SS are skin-homing T cells, and they exhibit Th2 and Treg phenotypes and highly express CCR4. Macrophage-derived chemokine or CCL22 and thymus and activation-regulated chemokine or CCL17 are two main ligands for CCR4. By interacting with CCL22 and CCL17, CCR4^+^ cells migrate and accumulate in the skin, inflammatory tissues, and tumors. The pathogenetic roles of CCR4 in inflammatory diseases and tumors have been widely studied. Studies have also found that CCR4 is a pathogenic driver in CTCL and involved in the development and progression of MF/SS (1). Thus, CCR4 has been considered to be a great therapeutic target for MF/SS.

The anti-CCR4 monoclonal antibody strategy has been successfully developed for patients with MF/SS. Mogamulizumab, a defucosylated anti-CCR4 antibody, can eliminate CCR4^+^ malignant T cells by antibody-dependent cellular cytotoxicity and has been approved by the FDA for patients with MF/SS (1–3). Clinical trials have shown that the overall response rates were significantly higher in patients with MF/SS when treated with mogamulizumab than when treated with vorinostat—the FDA-approved histone deacetylase inhibitor (4). But immune side effects related to CCR4^+^ Treg deletion have recently drawn attention (5–7). In fact, although mogamulizumab kills CCR4^+^ malignant T cells, it also depletes normal CCR4^+^ Tregs or other beneficial immune cells. Mogamulizumab-associated rash was reported in up to 68% of treated patients (8, 9), and severe side effects such as graft-versus-host disease are not uncommon (10, 11). Thus, more effective and less toxic therapeutic strategies to target CCR4 are needed. Small-molecule CCR4 antagonists are an excellent alternative, and they can selectively inhibit the function of CCR4 but not eradicate cells.

There are two classes of small-molecule CCR4 antagonists: class-I antagonists, a collection of lipophilic heteroarenes, bound to the intrahelical binding site of CCR4 and class-II antagonists, a range of aryl sulfonamides, bound to C-terminal helix-VII of CCR4. A few small-molecule CCR4 antagonists have been evaluated in diseases such as atopic dermatitis and cancers in which Th2 and Tregs play crucial roles in pathogenesis (12–15). However, the effects of small-molecule CCR4 antagonists on CTCL have not yet been studied. We hypothesize that small-molecule CCR4 antagonists can be a great alternative of CCR4-targeting therapy for patients with MF/SS in alleviating the side effects by more selectively inhibiting toward CCR4.

In this study, we selected C021 (16) as a representative of class-I CCR4 antagonists and AZD-2098 (17) as a representative of class-II CCR4 antagonists. The effects of C021 and AZD-2098 on CCR4 expression, cell chemotaxis, cell proliferation, cell apoptosis/cell cycle, and colony formation were assessed in MF-derived cell line (MJ) and SS-derived cell line (HuT 78) *in vitro*. The effects of C021 on tumor growth in CTCL xenograft mice *in vivo* were assessed as well.

## Materials and Methods

### Cells

Human CTCL cell lines, MJ (RRID: CVCL_1414) and HuT 78 (RRID: CVCL_0337) cells, were purchased from the ATCC. Cells were cultured with complete media, as previously described (18), with negative results for mycoplasma (tested on November 17, 2011, and March 29, 2023).

### CCR4 antagonists

C 021 dihydrochloride or C021 was purchased from Tocris Bioscience and stocked at 10 mmol/L in DMSO. AZD-2098 was purchased from Sigma Aldrich and stocked at 5 mmol/L in DMSO. The stocks were kept at −20°C until use. The working solutions were prepared on the day of assay.

### Flow cytometry analysis for CCR4 expression

Cells were first treated with C021 or AZD-2098 or vehicle control for 30 minutes. After washing with FACS buffer (PBS and 5% FBS), cells were incubated with CCR4-PE-CF594 antibody (IgG1, κ, Clone: 1G1, BD Biosciences, RRID: AB_2739215) for 20 minutes on ice. CCR4 expression on cell surfaces was then analyzed by flow cytometry on a Beckman Coulter Gallios Flow Cytometer. Cells that were stained with IgG1 conjugated with fluorophore (PE rat IgG1, κ isotype control; BD Biosciences, RRID: AB_395140) were included as control.

### qRT-PCR

MJ and HuT 78 cells were treated with C021 (IC_50_) or AZD-2098 (10 μmol/L) or DMSO for 12 hours. Total RNA was extracted, and a portion (1 μg) was treated with DNase I, followed by first-strand cDNA synthesis. Preformulated TaqMan primers and probes for CCR4 (Hs99999919_m1), IL-5 (Hs01548712_g1), IL-13 (Hs00174379_m1), and IL-32 (Hs00992441_m1) were used for qRT-PCR. GAPDH (Hs99999905_m1) was used as the endogenous control gene. The QuantStudio 3 Real-Time PCR System was used with the default “fast” protocol by the manufacturer (Applied Biosystems). The expression levels of CCR4, IL-5, IL-13, or IL-32 mRNA were quantitated on the basis of the Ct values and then normalized to GAPDH. Relative fold changes of these genes that were normalized to GAPDH were finally calculated (2, 19).

### Chemotaxis assay

Transwell plates (24-well, Corning) were used for the chemotaxis assay according to the manufacturer’s instructions with some modifications. Briefly, transwells were equilibrated with assay media (RPMI1640 and 1% BSA) for 1 hour at 37°C. After the assay media were removed, a series dilutions of chemokines (CCL22 or CCL17 at 0.1, 1, 10, 50, 100, and 1,000 μmol/L) were added into the bottom compartments. The cell suspensions (5 × 10^5^/well) were added into the upper compartments. The plates were then incubated at 37°C for 3 hours. The inserts were taken out after 3 hours, and the cells on the bottom compartments were counted. The concentrations of CCL22 or CCL17 that led to the highest chemotaxis were calculated for both MJ and HuT 78 cells and used in subsequent inhibitory chemotaxis assays. For the inhibitory chemotaxis assay, cells were first treated with C021 or AZD-2098 at different concentrations for 30 minutes, and then seeded in the upper compartments of the transwells. CCL21 (50 nmol/L) was used as chemokine control to confirm the specificity of inhibition of C021 or AZD-2098.

### Cell proliferation assay

Cells were seeded in 96-well plates (2.5 × 10^4^ cells/well) and treated with C021 or AZD-2098 (0.1, 0.5, 1.0, 5.0, 10.0, and 25.0 μmol/L) for 12 hours. Experiments were performed in triplicate. Cell viability was then determined using the CellTiter 96 AQueous One Solution Cell Proliferation Assay [an assay using 3-(4,5-dimethylthiazol-2-yl)-5-(3-carboxymethoxyphenyl)-2-(4-sulfophenyl)-2H-tetrazolium (MTS), Promega] as previously described (19). Absorbance was measured at 490 nm using the μQuant plate reader (BioTek).

### Soft agar colony formation assay

Soft agar colony formation assay was conducted to assess the effects of C021 on anchorage-independent growth ability as we previously reported with some modifications (18, 20). In brief, 0.7% soft agar in complete media was poured into wells first as a bottom layer and solidified. Then, an upper layer of 0.35% soft agar was added, which contained MJ and HuT 78 cells (15,000 cells/well in a six-well plate or 10,000 cells/well in a 12-well plate). The cells were treated with different doses of C021 twice a week or daily for 3 days per week for 2 or 3 weeks. At the end, the colonies were stained with 0.01% (v/v) of crystal violet. The stained colonies were counted.

### Flow cytometry analysis for apoptosis and cell cycle

Cells were incubated at 5 × 10^5^ cells/mL and treated with different doses of C021 or AZD-2098 for 12 hours at 37°C. The cells undergoing apoptosis were then analyzed using the annexin V-FITC detection kit-I (BD Pharmingen) by flow cytometry as previously described (19). For the cell-cycle analysis, cells were harvested after treatment with different concentrations and washed with cold PBS. Cells were fixed in cold 70% ethanol and kept at −20°C for 1 hour. After treating with RNase, DNase-free (Roche Diagnostics), cells were stained with propidium iodide (PI; 50 μg/mL, Sigma-Aldrich). The cell-cycle distribution, including sub-G_1_ populations were analyzed with a BD FACSCalibur flow cytometer as previously described (19).

### Proteome profiler human cytokine array

Proteome Profiler Human Cytokine Array Kit (R&D Systems) was used to analyze 36 cytokines, chemokines, and growth factors according to the manufacturer’s protocol as described previously (18). Briefly, cell lysates were prepared from MJ and HuT 78 cells treated with C021 (proliferation IC_50_) or vehicle controls for 12 hours. Protein was quantified using the Bradford assay. Lysates (200 μg per sample) were mixed with a cocktail of biotinylated detection antibodies and then incubated with the array membranes that are spotted in duplicate with capture antibodies to specific target proteins. Captured proteins are visualized using chemiluminescent detection reagents. Positive signals seen on the developed film and densities of positive cytokines detected were quantified using ImageJ software (NIH) as previously reported (18). The levels of cytokines, chemokines, and growth factors were compared between compound-treated cells and vehicle-only cells that were considered as onefold or 100%.

### Western blot

The equal cellular proteins (10 μg) extracted from treated or control cells were subjected to 4% to 20% Mini-PROTEAN TGX gel (Bio-Rad) electrophoresis and transferred onto nitrocellulose membranes (Whatman GmbH). The membranes were blocked in 5.0% milk in Tris-buffered saline with Tween 20 (TBST) (50 mmol/L Tris pH 7.5, 150 mmol/L NaCl, 0.05% Tween 20) for 1 hour at room temperature, then incubated with primary antibodies overnight at 4°C. Primary antibodies and dilutions that were used in this study are as follows: purified antihuman IL-32αβγδ antibody, KU32-52, 1:1,000 (RRID: AB_2124018); purified anti-mouse/human IL-5 antibody, TRFK5, 1:1,000 (RRID: AB_315325); and purified antihuman IL-13 antibody, JES10-5A2, 1:1,000 (RRID: AB_315196; BioLegend). After washing with TBST, the membranes were incubated with horseradish peroxidase–conjugated secondary antibodies for 1 hour at room temperature. Protein bands were visualized using the SuperSignal West Pico Chemiluminescence Substrate kit (Thermo Fisher Scientific). Equivalent loading of proteins was confirmed by Pan-Actin (D18C11, Cell Signaling Technology).

### Xenograft tumor formation and growth in NSG mice

All animal experiments in this study were approved by the Animal Care and Use Committee of the University of Texas MD Anderson Cancer Center (ACUF00002043-RN01). About 6- to 7-week-old female NSG mice (NOD.Cg-Prkdc^scid^ Il2rg^tm1Wjl/SzJ^, RRID: IMSR_JAX:005557) were obtained from The Jackson Laboratory. HuT 78 cells (5.0 × 10^6^/site, nine mice/group) were subcutaneously injected into NSG mice on day 1. The treatment started on day 2, and four groups of mice were treated with different doses of C021, route, and schedules: **Group 1 (G1)**—control, i.p., twice/week; **Group 2 (G2)**—5 mg/kg, i.p., twice/week; **Group 3 (G3)**—2 mg/kg, i.p., daily × thrice/week; **Group 4 (G4)**—5 mg/kg, s.c., twice/week. Tumor formation and growth were monitored twice a week or as needed. Tumor volumes were calculated using the formula: 1/2(length × width^2^) as previously described (18). On day 22, mice were sacrificed and tumors were collected and weighted. Tumors were processed and embedded in paraffin blocks. Sections were cut and stained with hematoxylin and eosin for histological evaluation. CCR4 and Ki67 expression in tumor tissues from different groups was analyzed by IHC. Primary antibodies and dilutions used for IHC were included: rabbit polyclonal anti-CCR4 antibody, 1:250, NBP1-86584 (RRID: AB_11025232; Novus Biologicals); rabbit monoclonal anti-Ki67 D2H10, 1:3,000, #9027S (Cell Signaling Technology).

### Statistical analysis

The differences in cell chemotaxis, cell proliferation, apoptosis, cell cycle, and colony formation in C021- or AZD-2098-treated cells were compared with that of untreated cells or vehicle controls. The differences in tumor volumes and IHC scores between four groups were also compared. Statistical analyses were performed using the GraphPad Prism software (RRID: SCR_002798, GraphPad Software, Inc.) for *t* test, χ^2^ test, or ANOVA as needed. The *P* value ≤ 0.05 was considered statistically significant, and the *P* value ≤ 0.01 was considered statistically very significant.

### Data availability

The data generated in this study are available upon request from the corresponding author.

## Results

### C021 and AZD-2098 exerted different effects on CCR4 expression in MJ and HuT 78 cells

**Figure 1:**
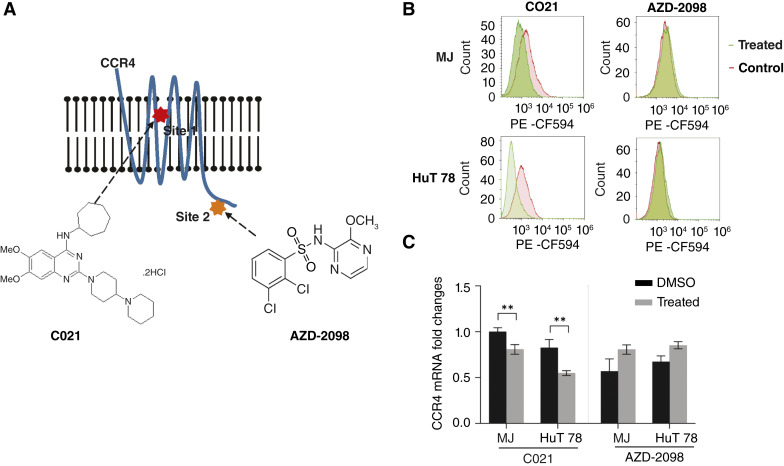
Two allosteric CCR4 antagonists and their effects on CCR4 expression in MJ and HuT 78 cells. **A,** Schematic cartoon of binding sites of two allosteric CCR4 antagonists: C021 binds to a transmembrane site on CCR4 (site 1, red), and AZD-2098 binds to intracellular or C-terminal site of CCR4 (site 2, orange); **B,** CCR4 expression on MJ and HuT 78 cells treated with C021 or AZD-2098 (green lines) or DMSO (red lines) was assessed by flow cytometry with anti-CCR4 antibody (CCR4-PE-CF594 antibody, IgG1, κ, Clone: 1G1); **C,** The expression of CCR4 mRNA in MJ and HuT 78 cells treated with C021 or AZD-2098 (gray) or DMSO (black) was assessed by qRT-PCR. The fold changes of CCR4 expression are plotted in pairs of treated vs. DMSO. Paired *t* test, **, *P* < 0.01.

It is known that small-molecule CCR4 antagonists could selectively inhibit function of CCR4. In this study, we studied two known CCR4 allosteric antagonists with different binding sites on CCR4. As shown in Fig. 1A, the first CCR4 antagonist is C021, which binds to a transmembrane site on CCR4 (site 1) allowing for internalization of CCR4. The other CCR4 antagonist is AZD-2098, which binds to an intracellular or C-terminal site of CCR4 (site 2) in which no internalization happens. To confirm the effects of the two CCR4 antagonists on CCR4 expression, MJ and HuT 78 cells were treated with C021 (3.21 μmol/L—MJ cells and 5.98 μmol/L—HuT 78 cells) or AZD-2098 (10 μmol/L for both cell lines) or vehicle control for 30 minutes, then CCR4 expression was assessed by flow cytometry. As per results, CCR4 expression was decreased in MJ and HuT 78 cells treated with C021 [Fig. 1B (green)] in comparison with vehicle control [Fig. 1B (red)]. However, little changes were seen in MJ or HuT 78 cells treated with AZD-2098.

We also assessed mRNA expression of CCR4 in MJ and HuT 78 cells treated with C021 or AZD-2098 for 12 hours by qRT-PCR. As shown in Fig. 1C, the expression of CCR4 mRNA was decreased in both MJ and HuT 78 cell lines treated with C021 (paired *t* test, *P* < 0.01). However, the expression of CCR4 mRNA was slightly increased in MJ cells and HuT 78 cells treated with AZD-2098. These results suggest that C021 and AZD-2098 exert different effects on CCR4 expression in MJ and HuT 78 cells.

### C021 and AZD-2098 inhibited chemotaxis to CCL17 and CCL22 in MJ and HuT 78 cells

**Figure 2:**
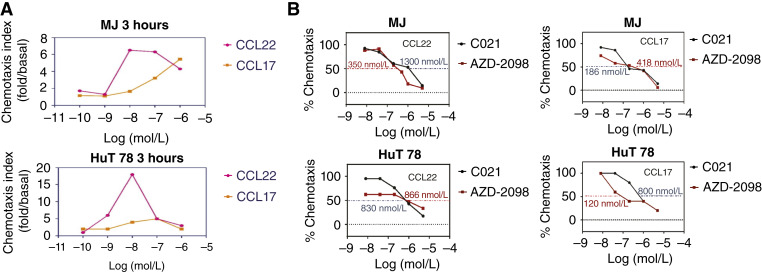
Chemotaxis of MJ and HuT 78 cells to CCL22 and CCL17 and inhibitory effects of CCR4 antagonists on chemotaxis. **A,** Transwell plates were used for the chemotaxis assay. Chemotaxis of MJ and HuT 78 cells to series doses of CCL22 or CCL17 [log(M)] was assessed after 3 hours, and the data are plotted. **B,** For the inhibitory chemotaxis assay, cells were first treated with series doses of C021 or AZD-2098 for 30 minutes and then used for the chemotaxis assay. The dose-dependent inhibition of C021 or AZD-2098 on chemotaxis to CCL17 (100 nmol/L) and CCL22 (10 nmol/L) is plotted for MJ and HuT 78 cells, respectively.

Before assessing the effects of CCR4 antagonists on cell chemotaxis to CCL17 and CCL22, we first conducted chemotaxis assays in MJ and HuT 78 cells at a wide range of concentrations of CCL22 or CCL17. The cells were plated on the top transwells, and different concentrations of CCL22 or CCL17 were plated on the bottom wells. After 3-hour incubation, cell numbers in the bottom wells were counted. As shown in Fig. 2A, both MJ and HuT 78 cells showed chemotaxis to CCL22 or CCL17, and the sensitivity of chemotactic response to CCL22 was higher than it was to CCL17. The highest chemotaxis to CCL22 in both MJ and HuT 78 cells was observed at 10 nmol/L, and it then declined regardless of the increasing concentrations of CCL22. The highest chemotactic response to CCL17 was seen at 100 nmol/L in HuT 78 cells, and MJ cells showed continuous increased chemotaxis beyond 100 nmol/L. These results confirm that MJ and HuT 78 cells exhibit chemotaxis to CCL22 and CCL17, and they may be more sensitive to CCL22 than to CCL17.

We then assessed the effects of C021 or AZD-2098 for chemotaxis to CCL22 (at 10 nmol/L) or CCL17 (at 100 nmol/L) in MJ and HuT 78 cells. The cells were first treated with different concentrations of C021 or AZD-2098 for 30 minutes, and then plated on the top transwells. After 3-hour incubation, cells migrated to the bottom were counted. As shown in Fig. 2B, dose-dependent inhibition of chemotaxis to CCL22 or CCL17 by C021 or AZD-2098 was seen in MJ and HuT 78 cells. The concentrations for 50% of chemotaxis inhibition (IC_50_) were calculated for the two compounds in two cell lines and are summarized in Table 1. From Table 1, it can be seen that C021 has stronger inhibition on chemotaxis to CCL17 (IC_50_ = 186 nmol/L) than to CCL22 (IC_50_ = 1,300 nmol/L) in MJ cells. AZD-2098 has stronger inhibition on chemotaxis to CCL17 (IC_50_ = 120 nmol/L) than to CCL22 (IC_50_ = 866 nmol/L) in HuT 78 cells. Our results suggest that both C021 and AZD-2098 exert inhibitory effects on chemotaxis to CCL17 and to CCL22 in MJ and HuT 78 cells.

**Table 1:** IC_50_ of CCR4 antagonists on chemotaxes and cell proliferation in MJ and HuT 78 cells

	Cell lines	Chemokine	C021 (nmol/L)	AZD-2098 (nmol/L)
Chemotaxis	MJ	CCL22	1,300	350
		CCL17	186	418
	HuT 78	CCL22	830	866
		CCL17	800	120
Proliferation	MJ	**—**	3,210	N/A
	HuT 78	**—**	5,980	N/A

Abbreviation: N/A, not available.

### C021 inhibited cell proliferation and colony formation in MJ and HuT 78 cells

**Figure 3:**
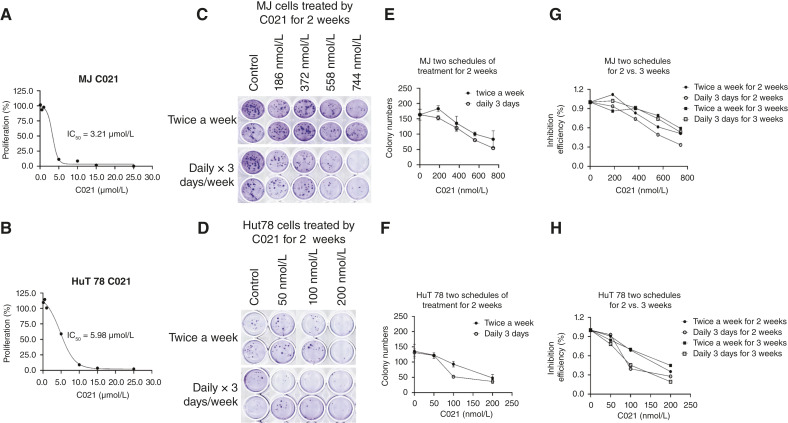
Effects of C021 on cell proliferation and colony formation in MJ and HuT 78 cells. **A** and **B,** MJ and HuT 78 cells were seeded in 96-well plates and treated with series doses of C021 or vehicle controls for 12 hours. The cell viability was then determined using the CellTiter 96 AQueous One Solution Cell Proliferation Assay (MTS). The data are analyzed and plotted. IC_50_ was calculated for MJ and HuT 78 cells. **C** and **D,** Soft agar colony formation assay was used to assess the effects of C021 on anchorage-independent growth ability. MJ and HuT 78 cells were treated with different doses of C021 twice a week or daily for 3 days per week for 2 weeks. The colonies were stained with 0.01% (v/v) of crystal violet and pictured at the end. The same control wells were used for each treatment schedule. **E** and **F,** The numbers of colonies (mean ± SD) of MJ and HuT 78 cells with two different treatments for 2 weeks are counted and plotted. **G** and **H,** The dose-dependent inhibitions of C021 on colony formation (%) in MJ and HuT 78 cells with two different treatments for 2 and 3 weeks are plotted.

We next assessed the effects of CCR4 antagonists on cell proliferation. MJ and HuT 78 cells were treated with different concentrations of C021 or AZD-2098 for 12 hours, and the cell proliferation were then assessed using MTS assay. The cell proliferation rates in cells treated with vehicle controls were considered as 100%. The concentrations of CCR4 antagonists for 50% of cell proliferation inhibition (IC_50_) were calculated for both cell lines. As per results, IC_50_ of C021 for MJ cells was 3.21 μmol/L and that of C021 for HuT 78 cells was 5.98 μmol/L (Fig. 3A and B; Table 1). However, no inhibition was observed in MJ or HuT 78 cells treated with AZD-2098 at any concentrations.

We next only tested the effects of C021 on colony formation in cell lines. Soft agar colony formation assay was used to assess the anchorage-independent growth ability in MJ and HuT 78 cells according to literature with some modifications (20). C021 was added twice a week or daily for consecutive 3 days a week for 2 or 3 weeks. As shown in Fig. 3C and D, more and larger colonies were observed in MJ cells than in HuT 78 cells, with the same numbers of seeding cells. Overall, there were dose-dependent inhibitions in both cell lines. In comparison, among different treatment schedules, cells treated with daily schedule had less colonies than the ones treated with twice-a-week schedule for either 2- or 3- week periods (Fig. 3C–H). Our results suggest that C021 exerts potent inhibitory effects not only on cell proliferation but also on colony formation in MJ and HuT 78 cells. The different treatment schedules affect the inhibiting effects on colony formation.

### C021 induced cell apoptosis and cell-cycle arrest in MJ and HuT 78 cells

**Figure 4:**
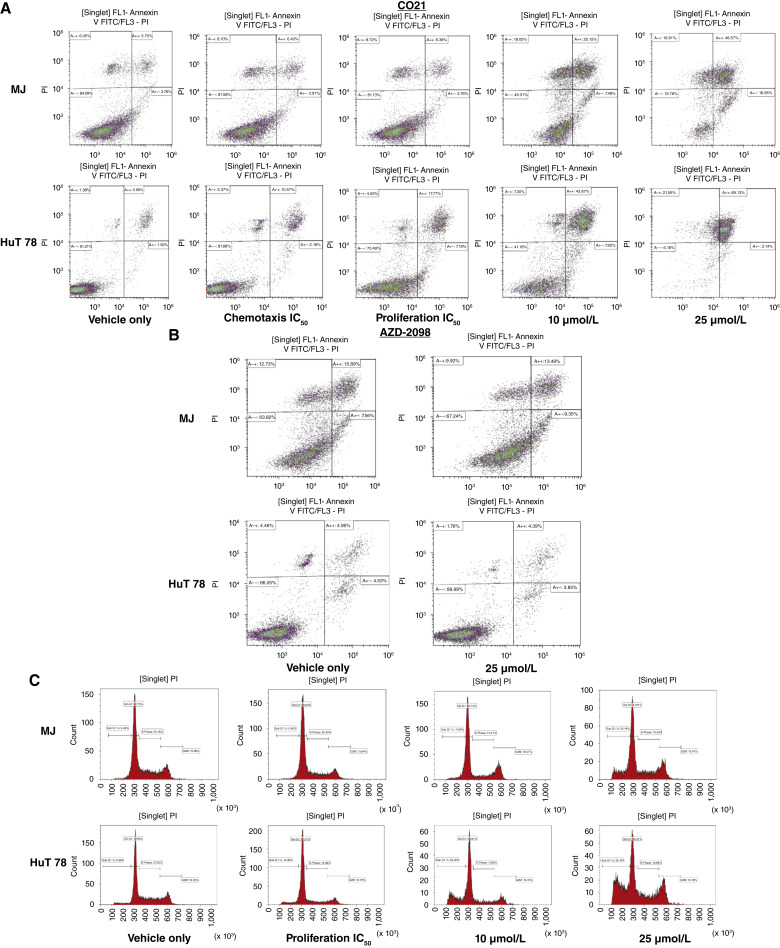
Effects of C021 and AZD-2098 on apoptosis and cell cycle in MJ and HuT 78 cells. **A,** MJ and HuT 78 cells (5 × 10^5^/mL) were treated with different doses of C021 or vehicle control for 12 hours. Apoptotic cells were assessed by flow cytometry using the annexin V–FITC Detection Kit. Dot plots from flow cytometry analysis for MJ and HuT 78 cells are presented. **B,** MJ and HuT 78 cells (5 × 10^5^/mL) were treated with AZD-2098 at 25.0 μmol/L or vehicle control for 12 hours, and dot plots from flow cytometry analysis are presented. **C,** MJ and HuT 78 cells (5 × 10^5^/mL) are treated with different doses of C021 (proliferation IC_50_, 10.0, and 25.0 μmol/L) or vehicle control for 12 hours. Cell-cycle analysis was performed by assessing DNA content distribution by flow cytometry with propidium iodide (PI) staining. Histograms for cell cycle (sub-G_1_, G_0_/G_1_, S, and G_2_/M) are present.

We next further assessed the effects of C021 and AZD-2098 on induction of apoptosis and cell-cycle arrest in MJ and HuT 78 cells. Overall, dose-dependent apoptotic cell inductions were found in MJ and HuT 78 cells treated with C021 (Fig. 4A). Of note, HuT 78 cells had increased apoptotic cells when cells treated with chemotaxis IC_50_ (800 nmol/L) and apoptotic cells continued to increase along with increasing doses of C021 (proliferation IC_50_—5.98 μmol/L, 10 μmol/L, and 25 μmol/L). Of interest, MJ cells had no apoptotic cell increase until cells were treated with 10 μmol/L of C021. There were no cells undergoing apoptosis in MJ and HuT 78 cells when they were treated with AZD-2098, even with the highest dose of 25 μmol/L (Fig. 4B).

We also assessed the effects of C021 on cell cycles. Cells were treated with three different concentrations of C021 (proliferation IC_50_: 10 μmol/L and 25 μmol/L). As shown in Fig. 4C, dose-dependent sub-G_1_ population increases were observed in both MJ and HuT 78 cells with C021 treatment. Like apoptosis induction, HuT 78 cells showed sub-G_1_ cell portion increase starting at cells treated with proliferation IC_50_, and dramatically increased at 10 and 25 μmol/L. But there was no sub-G_1_ population increase in MJ cells treated with proliferation IC_50_ until 10 μmol/L of C021. These results suggest that HuT 78 cells are more sensitive to C021 than MJ cells in apoptosis induction and cell-cycle arrest.

### C021 downregulated the expression of IL-5 in MJ and HuT 78 cells

**Figure 5:**
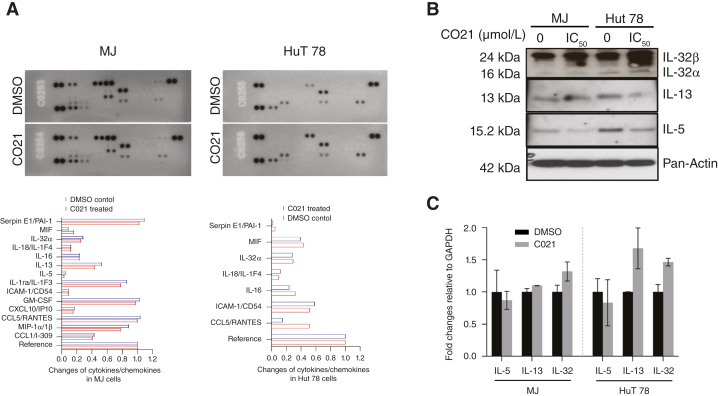
Effects of C021 on cytokines and chemokines in MJ and HuT 78 cells. **A,** Proteome Profiler Human Cytokine Array Kit is used to analyze cytokines and chemokines. Cell lysates (200 μg per sample) from MJ and HuT 78 cells treated with C021 (proliferation IC_50_ for 12 hours) or DMSO were used for the assay. The positive signals on developed film are quantified and plotted for C021-treated cells and DMSO-treated cells, which are considered to be onefold or 100%. **B,** Cell lysates from MJ and HuT 78 cells treated with C021 or DMSO were also used for Western blot analysis to confirm IL-32, IL-5, and IL-13. The cellular proteins (10 μg) were fractionated by 8% to 12% SDS-PAGE, transferred onto nitrocellulose membranes and subjected to enhanced chemiluminescence (Amersham) detection analysis. The equivalent loading of proteins in each well was confirmed by Pan-Actin. **C,** The expression of IL-5, IL-13, and IL-32 mRNA in MJ and HuT 78 cells treated with C021 or AZD-2098 (gray) or DMSO (black) was assessed by qRT-PCR. The fold changes of IL-5, IL-13, and IL-32 expression normalized to GAPDH are plotted in pairs of treated vs. DMSO.

To study the immunomodulation effects of C021 on CTCL cell lines, the levels of 36 cytokines, chemokines, and growth factors were assessed simultaneously using Proteome Profiler Human Cytokine Array. The changes of cytokine expression in treated cells were compared with cells treated with vehicle controls, which were considered to be onefold or 100%. As shown in Fig. 5A, 14 cytokine expressions showed measurable changes in treated MJ cells, and 11 of them were downregulated. Two Th2 cytokines, IL-5 and IL-13, were among them. Only seven cytokine expressions had measurable changes in treated HuT 78 cells. Of interest, these seven cytokines were among the cytokines affected in MJ cells. Three of them (MIF, IL18/IL1F4, and ICAM-1/CD54) showed similar changes (downregulation), whereas the other four (Serpin E1/PAI-1, IL-32α, IL16, and CCL5/RANTES) had different changes.

We have previously reported that IL-32 was upregulated in CTCL cell lines and primary Sézary cells (21) and speculate its possible oncogenic roles in pathogenesis of CTCL. Here by cytokine array, downregulated IL-32α expression was seen in treated MJ cells but a little upregulated IL-32α was observed in treated HuT 78 cells. To further confirm these findings, Western blots were performed for IL-5, IL-13, and IL-32 in both cell lines. As per results, the levels of IL-5 were decreased in both MJ and HuT 78 cell lines treated with C021. The decreased levels of IL-13 were clearly seen in HuT 78 cell lines treated with C021 but not in MJ cells. However, increased IL-32α and IL-32β levels were seen in HuT 78 cells and in MJ cells (Fig. 5B).

We further assessed mRNA expression of IL-5, IL-13, and IL-32 using qRT-PCR. As shown in Fig. 5C, IL-5 expression decreased in both MJ and HuT 78 cell lines treated with C021. Conversely, IL-13 and IL-32 expression increased in both cell lines. These results suggest that C021 exerts immunomodulating effects on CTCL cells, and it downregulates IL-5 but upregulates IL-32.

### C021 inhibited tumor growth in CTCL xenograft mouse models

**Figure 6:**
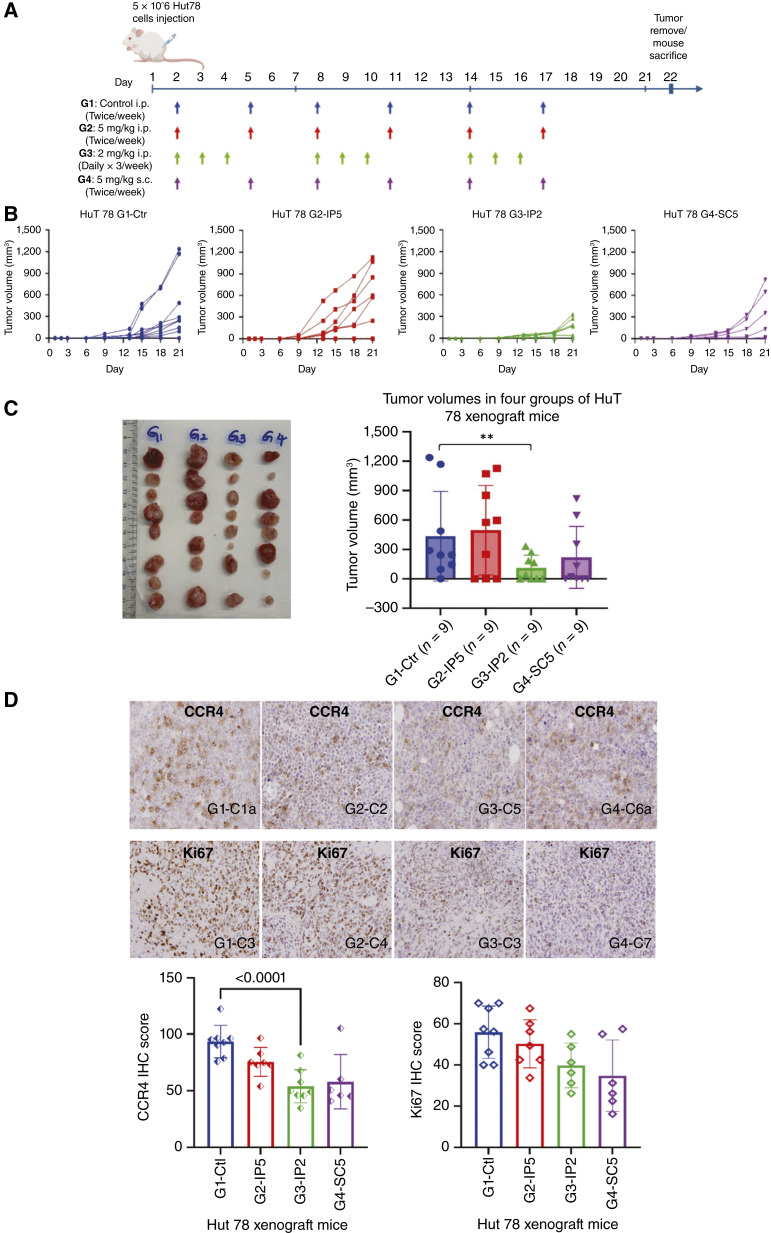
Effects of C021 on tumor formation and growth in CTCL xenograft mice. **A,** Schedules of C021 treatment in xenograft mice. HuT 78 cells (5.0 × 10^6^/site, nine mice/group) were subcutaneously injected into NSG mice on day 1. The treatment started on day 2, and four groups of mice were treated with different doses, routes, and schedules as indicated: **G1**—control (Ctl), i.p., twice/week; **G2**—5 mg/kg, i.p., twice/week; **G3**—2 mg/kg, i.p., daily × thrice/week; **G4**—5 mg/kg, s.c., twice/week. **B,** Tumor formation and growth were monitored, and tumor volumes (mm^3^) over 3 weeks are plotted for **G1–4**, respectively. **C,** The tumor tissues collected on day 22 are displayed, and the final tumor volumes (mm^3^) are plotted for all mice in four groups. **D,** Tumors tissue sections were stained for CCR4 and Ki67 protein expression by immunohistochemistry. Representative sections from **G1** to **G4** are presented for CCR4 (top) and Ki67 (bottom). IHC scores for the four groups are plotted for CCR4 and Ki67 expression. **, ANOVA test: *P* < 0.0001, statistically highly significant.

We next conducted *in vivo* experiments in CTCL xenograft mouse models. As indicated in Fig. 6A, mice from the four groups (nine mice/group) were include in this study and treated with different schedules (twice a week or daily × 3 days a week), different doses of C021 (5 mg/kg or 2 mg/kg), and different routes (i.p. and s.c.). HuT 78 cells (5 × 10^6^/site) were injected subcutaneously into NSG mice on day 1. The treatment started on day 2 and continued for 3 weeks. Tumor formation and growth were monitored twice a week or as needed. Mice were sacrificed on day 22 and tumors were collected for histopathology by hematoxylin and eosin staining and IHC for CCR4 and Ki67 expression.

As per results, tumors were observed/detected on days 9 to 13, and tumors in **G3** mice (2 mg/kg, i.p., daily × thrice/week) grew the slowest among the four groups in general (Fig. 6B). Tumors in **G4** mice (5 mg/kg, s.c., twice/week) grew slower than those in **G1** (control, i.p., twice/week) and **G2** (5 mg/kg, i.p., twice/week). There was no tumor formation in one of nine mice in **G1**, and two of nine mice in **G2**, **G3**, and **G4**. The final tumor volume on average in **G3** mice was the smallest (38.8 mm^3^, ANOVA, *P* < 0.001) among the four groups. The final tumor volume on average in **G4** (83.1 mm^3^) was smaller than those in **G1** (239.8 mm^3^) and **G2** (307.3 mm^3^; Fig. 6C).

By IHC, CCR4 expression on cell membrane was observed in most cells of tumor sections from all groups as shown in the top panel of Fig. 6D. The average CCR4 IHC scores in **G3** were lowest among four groups. Tumor sections in **G4** had lower IHC scores than those in **G1** and **G2**. By IHC, Ki67 nuclear expression was seen in a majority of the cells of the tumor sections [Fig. 6D (bottom)]. Of note, the average Ki67 IHC score in **G4** was the lowest among the four groups. The Ki67 IHC scores in **G3** were lower than those in **G2** and **G1**.

Our *in vivo* results suggest that C021 has an inhibitory effect on tumor growth in CTCL xenograft mouse models. Our results also suggest that C021 administered daily may be more effective in inhibiting tumor growth than when administered twice a week, and the subcutaneous injection may be a better route than the intraperitoneal route of administration.

## Discussion

In summary, our study found that both C021 and AZD-2098 had inhibitory effects on cell chemotaxis to CCL17 and CCL22 in MJ and HuT 78 cells. Only C021 inhibited cell proliferation, induced cell apoptosis and cell-cycle arrest, and decreased colony formation in MJ and HuT 78 cells *in vitro*. Also, only C021 inhibited tumor growth in CTCL xenograft mice *in vivo*. These findings suggest that C021 has more inhibitory effects than AZD-2098 on CTCL cells and may have potentials for clinical implications.

As mentioned previously, C021 and AZD-2098 belong to two different classes of small-molecule CCR4 antagonists. The different effects on CTCL cells between C021 and AZD-2098 may be attributed by their different binding sites to CCR4 and subsequent signaling. C021, a class-I CCR4 antagonist, binds to the lipid bilayer part of CCR4 which triggers the internalization of CCR4 and leads to decreased CCR4 on cell membranes. AZD-2098, a class-II CCR4 antagonist, binds to the intracellular region of CCR4, which does not activate the internalization of CCR4 and has no change in CCR4 expression on the cell surface. Our results confirm the different CCR4 expression on treated cells between C021 and AZD-2098 by flow cytometry and qRT-PCR. Given that CCR4 mRNA levels remained low after 12 hours of C021 treatment, it suggests that C021 has a sustained impact on CCR4 expression, possibly due to receptor internalization or ongoing signaling that downregulates transcription. The slight increase in CCR4 mRNA after AZD-2098 treatment could indicate a compensatory mechanism or differential regulation between the two compounds. Thus, we hypothesize that the transmembrane binding and reduction of CCR4 expression by C021 may attribute to its antitumor effects.

It is known that the interaction of CCR4 with its ligands CCL22 and CCL17 plays an important role in skin-homing of CCR4^+^ T cells and the development and progression of MF/SS (1). Our results in this study confirm that MJ and HuT 78 cells are chemotactic to both CCL22 and CCL17. In addition, we found that CCL22 is a more potent chemoattractant to MJ and HuT 78 cells than it is to CCL17. Both MJ and HuT 78 cells reached the chemotaxis peaks at 10 nmol/L of CCL22. Instead, HuT 78 cells reached the chemotaxis peak at 100 nmol/L of CCL17, and MJ cells did not reach the peak at 1,000 nmol/L of CCL17. Both C021 and AZD-2098 had potent inhibition of chemotaxis to CCL22 and CCL17, although two compounds showed differences in other cellular effects.

In this study, we observed that HuT 78 cells (SS-derived cell line) were more sensitive to apoptosis induction by C021 compared with MJ cells (MF–derived cell line). This finding aligns with clinical data from patients with MF/SS treated with mogamulizumab, wherein patients with SS demonstrated a clinical response rate of 47.1% when compared with a 28.6% response rate in patients with MF (2, 3). Additionally, patients with B1 (5% Sézary cells) and B2 (≥ 1000 Sézary cells/µL) blood involvement showed a high blood response rate of 94.7% (3). The difference in clinical responses between patients with SS and MF may partly be due to the intravenous administration of mogamulizumab, which exposes Sézary cells in the blood to high concentrations of anti-CCR4 antibodies. However, the mechanisms underlying the differing sensitivities to small-molecule CCR4 antagonists between HuT 78 and MJ cell lines remain unknown, as both were treated identically in our *in vitro* experiments. Further studies are needed to elucidate these differences.

Our results from CTCL xenograft mice indicate that not only the doses but also the treatment schedules and administration routes affected the effectiveness of C021 in inhibiting tumor growth. C021 administered intraperitoneally daily was the most effective treatment regimen in inhibiting tumor growth among the four treatments. C021 administered subcutaneously was more effective in inhibiting tumor growth than when administered intraperitoneally at the same dose. Thus, future studies to test and optimize the treatment regimens are needed.

Our study has a couple of limitations that should be noted. First, we only tested one CCR4 antagonist in each class (C021 and AZD-2098). Although our findings provide valuable insights, further studies involving a broader range of CCR4 antagonists could help confirm and expand upon these results. Second, the CTCL xenograft mouse model used in this study reflects human pathogenesis to a limited degree. To fully understand the therapeutic potential of CCR4 antagonists, additional studies using human tissues and clinical investigations will be necessary.

This is the first study to investigate the effects of small-molecule CCR4 antagonists on CTCL. Our results demonstrate that C021, a class-I CCR4 antagonist, exerts multiple antitumor effects on CTCL cells *in vitro* and inhibits tumor growth in CTCL xenograft mice *in vivo*, whereas AZD-2098, a class-II CCR4 antagonist, does not show the same efficacy. The differences observed between these two CCR4 antagonists may provide valuable insights for designing future studies and selecting more effective CCR4-targeting compounds. Our findings suggest that small-molecule CCR4 antagonists could be a promising alternative therapeutic strategy for targeting CCR4^+^ CTCL cells. Unlike anti-CCR4 antibodies like mogamulizumab, which may cause side effects related to the deletion of CCR4^+^ Treg cells, small-molecule CCR4 antagonists may inhibit CCR4 function without eradicating cells, potentially reducing or minimizing these side effects.
